# The rate of ward to intensive care transfer and its predictors among hospitalized COPD patients, a retrospective study in a local tertiary center in Saudi Arabia

**DOI:** 10.1186/s12890-023-02775-z

**Published:** 2023-11-22

**Authors:** Abdallah Y Naser, Mohammad Saleh Dairi, Hassan Alwafi, Deema Sami Ashoor, Sami Qadus, Abdulelah M Aldhahir, Abdullah A Alqarni, Wael Aly Elrefaey, Sultan Qanash, Waleed Hafiz, Jaber S. Alqahtani, Rakan Ekram, Amjad Abuirmeileh, Anan S. Jarab, Omaima Ibrahim Badr

**Affiliations:** 1 Department of Applied Pharmaceutical Sciences and Clinical Pharmacy, Faculty of Pharmacy, Isra University, Amman, Jordan; 2 Department of Medicine, College of Medicine, Umm Al-Qura University, Makkah, 21955 Saudi Arabia; 3 Pharmacology and Toxicology Department, Faculty of Medicine, Umm Al-Qura University, Makkah, 21955 Saudi Arabia; 4 Department of Pharmacy, Faculty of health sciences, American University of Madaba, Madaba, Jordan; 5 Respiratory Therapy Department, Faculty of Applied Medical Sciences, Jazan University, Jazan, Saudi Arabia; 6 Department of Respiratory Therapy, Faculty of Medical Rehabilitation Sciences, King Abdulaziz University, Jeddah, Saudi Arabia; 7 Department of Pulmonary Medicine, Al Noor Specialist Hospital, Mecca, Saudi Arabia; 8 Department of Internal Medicine, National Guard Hospital, Jeddah, Saudi Arabia; 9 Department of Respiratory Care, Prince Sultan Military College of Health Sciences, Dammam, Saudi Arabia; 10 School of Public Health and Health Informatics, Umm Al-Qura University, Mecca, Saudi Arabia; 11 Faculty of Pharmacy, Middle East University, Amman, Jordan; 12 College of Pharmacy, AL Ain University, P.O. Box 112612, Abu Dhabi, United Arab Emirates; 13 AAU Health and Biomedical Research Center, Al Ain University, P.O. Box 112612, Abu Dhabi, United Arab Emirates; 14 Department of Clinical Pharmacy, Faculty of Pharmacy, Jordan University of Science and Technology, Irbid, 22110 Jordan; 15 Department of Chest Medicine, Faculty of Medicine, Mansoura University, Mansoura, 35516 Egypt

**Keywords:** Admissions, Hospital, COPD, ICU, Saudi Arabia

## Abstract

**Objective:**

To investigate the prevalence of intensive care unit (ICU) admission and its predictors among hospitalized chronic obstructive pulmonary disease (COPD) patients.

**Methods:**

An observational retrospective study was conducted. All patients with a confirmed diagnosis of COPD according to the GOLD guidelines between 28 and 2020 and 1 March 2023 at Al-Noor Specialist Hospital were included in this study. Patients were excluded if a preemptive diagnosis of COPD was made clinically without spirometry evidence of fixed airflow limitation. Descriptive results were presented as frequency (percentage) for categorical variables and mean (SD) for continuous variables and to estimate prevalence of ICU admission. Predictors of ICU admission among hospitalized COPD patients were determined using logistic regression analysis. A SPSS (Statistical Package for the Social Sciences) version 25 was used to perform all statistical analysis.

**Results:**

A total of 705 patients with COPD were included in this study. The mean age was 65.4 (25.3) years. Around 12.4% of the hospitalized patients were admitted to the ICD. Logistic regression analysis identified that older age (OR; 1.92, (1.41–2.62)), smoking (OR; 1.60 (1.17–2.19)), and having specific comorbidities (Hypertension (OR; 1.98 (1.45–2.71)), Diabetes mellitus (OR; 1.42 (1.04–1.93)), GERD (OR; 2.81 (1.99–3.96)), Ischemic heart disease (OR; 3.22 (2.19–4.75)), Obstructive sleep apnea syndrome (OR; 2.14 (1.38–3.33)), stroke (OR; 4.51 (2.20–9.26))) were predictors of ICU admissions among patients with COPD.

**Conclusions:**

Our study found that a step-up approach to inpatient COPD management requires admission to the ICU in 12.4%, for which age, smoking status, cardiovascular, and stroke were important predictors. Further clinical research is needed to provide a validated model that can be incorporated into clinical practice to monitor this patient population during their admission and identify at-risk individuals for early transfer to higher acuity settings and intensive care units.

## Background

Chronic obstructive pulmonary disease (COPD) is a common and treatable disorder that results in narrowing of the airways and decreased lung recoil as a result of chronic inflammation caused by long-term exposure to irritating particles or gases, most often cigarette smoke. [1–3] This disease often presents with symptoms of cough, dyspnea, and sputum production. [1] Significant disease can eventually results in respiratory failure [1, 4]. COPD prevalence has been steadily climbing globally over the last two decades; it had a prevalence of 8.9% in 2006 that has increased to 13.1% in 2019 [5–8]. Currently, it is the third most frequent cause of morbidity and mortality worldwide [1].

Although COPD is thought of primarily as a lung disease, recent advances in clinical and translational researches have confirmed its complex phenotyping as well as its associated extra-pulmonary consequences [9]. COPD is characterized by an obstruction of airflow that is not totally reversible. Emphysematous degradation of the parenchyma causes remodeling of the small-airway compartment and loss of elastic recoil, resulting in progressive reduction in forced expiratory volume in one second (FEV1), inadequate lung emptying on expiration, and subsequent static and dynamic hyperinflation. [10] Airway epithelial cell damage can result in a non-specific inflammatory response. [11] COPD patients are frequently at greater risk of comorbidities such as cardiovascular diseases, diabetes, osteoporosis, lung cancer, and psychological disorders such as anxiety and depression [12].

COPD is often clinically suspected in individuals who have relevant symptoms and risk factors. nevertheless, spirometry is mandatory to confirm the diagnosis [1]. Patient education, preventative treatment that focuses on avoiding irritants and immunizations, medications, pulmonary rehabilitation, and oxygen therapy are all part of COPD management [9]. COPD patients may suffer complications such as acute exacerbation, acute and/or chronic respiratory failure, pulmonary hypertension, cor pulmonale, weight loss, and bacterial infections as a result of poor treatment adherence, adverse responses to glucocorticoids, or progression of their underlying lung disease [13].

Comorbidities, past hospitalizations, and a longer hospital stay are associated with early readmission and can potentially predict disease severity [14, 15]. Comorbidities coexisting with COPD imply a more severe course of the disease, with more frequent exacerbations and hospitalizations [2, 9, 16]. People who have two or more exacerbations per year have a lower quality of life, a faster loss of lung function, more hospitalizations, and a higher mortality rate. [17–20]. A longer hospital stay increases the likelihood of hospital-acquired complications and further deterioration of the patient’s condition [21, 22].

The available literature provides a variety of prevalence rates for ICU admissions associated with acute exacerbations of chronic obstructive pulmonary disease (AECOPD) that necessitate hospitalization, ranging from 2 to 19% [23]. A previous study conducted in Saudi Arabia assessed the outcomes of patients hospitalized with exacerbation of COPD within the hospital setting, specifically focusing on in-hospital and ICU outcomes [24]. This study revealed that a total of 33% of the patients who were admitted required mechanical ventilation. Furthermore, the median duration of mechanical ventilation was determined to be 2.6 days, with a range spanning from 1 to 42 days. The median durations of the ICU and hospital stays were 3 (range: 1–40) and 9 (range: 2–43) days, respectively [24]. The mortality rate in the ICU was found to be 6%, whereas the mortality rate within the hospital setting was seen to be 11%. Higher hospital mortality was observed in cases where there was a low Glasgow Coma Scale at admission, intubation was required, mechanical ventilation was prolonged, the individual was a current smoker, a tracheostomy was performed, cardiac arrest occurred, or severe renal failure developed [24]. The length of hospital stay and comorbidities among COPD patients have previously been described. However, the predictors and prevalence of ICU admission in hospitalized COPD patients have not been well elucidated in prior studies in the region. Therefore, the objective of this study is to investigate the prevalence of ICU admission and its predictors among hospitalized COPD patients.

## Methods

### Study design and study settings

A retrospective cross-sectional study was conducted in a local tertiary center. All patients with a confirmed COPD diagnoses between 28 and 2020 and 1 March 2023 at Al-Noor Specialist Hospital electronic medical records were included in this study. Al-Noor Specialist Hospital in Mecca, Saudi Arabia, is a tertiary center with a capacity of 500-bed. It delivers tertiary care services to all residents and visitors of Mecca region of Saudi Arabia and it is part of the Ministry of Health Services [25].

### Study population

Patients with a confirmed diagnosis of COPD according to the GOLD guidelines were included in this study [26]. Patients were excluded if a preemptive diagnosis of COPD was made clinically without spirometry evidence of fixed airflow limitation. Patients with COPD were identified via both paper and electronic records using a unique medical record number (MRN) for each patient.

### Sample size calculation

To estimate the sample size needed for this study, we used the World Health Organization (WHO) recommendations for the minimal sample size needed for a prevalence study. Using a confidence interval of 95%, a standard deviation of 0.5, and a margin of error of 5%, the required sample size was 385 participants from each study population [27].

### Statistical analysis

Descriptive results were presented as frequency (percentage) for categorical variables and mean (SD) for continuous variables and to estimate prevalence of ICU admission. Predictors of ICU admission among hospitalized COPD patients were determined using logistic regression analysis. A confidence interval of 95% (p < 0.05) was applied to represent the statistical significance of the results and the level of significance was assigned as 5%. SPSS (Statistical Package for the Social Sciences) version 25.0 software (SPSS Inc.) was used to perform all statistical analysis.

## Results

### Patients’ baseline characteristics

A total of 705 patients with COPD were included in this study. More than half of them were males (60.3%). The mean age was 65.4 (25.3) years, and the mean BMI was 29.2 (6.8) kg/cm^2^. Greater a little over one-third the patients were current smokers. A total of 7.8% received home care. The most common comorbidities among the patients were diabetes mellitus and hypertension accounting for 40.9% and 40.7%, respectively, Table 1.

**Table 1:** Patients’ baseline characteristics

Variable	Frequency	Percentage
**Gender**		
Males	425	60.3%
**Mean age** (Standard deviation (SD)) years	65.4 (25.3) years	
**Mean Body Mass Index (BMI)** (Standard deviation (SD)) kg/cm^2^	29.2 (6.8) kg/cm^2^	
**Smoking status**		
Non-smoker	182	25.8%
Ex-smoker	258	36.5%
Current smoker	265	37.5%
Long-Term Home Care Service		
Yes	55	7.8%
**Mean FEV1**	62.9 (17.3)	
**Mean FEV1/FVC ratio**	57.5 (10.3)	
**Comorbidities**		
Diabetes mellitus	288	40.9%
Hypertension	287	40.7%
Gastroesophageal diseases (GERD)	186	26.4%
Ischemic heart disease	135	19.1%
OSAS	92	13.0%
Heart failure	65	9.2%
Chronic kidney disease	40	5.7%
Stroke	38	5.4%
Connective tissue diseases	23	3.3%
Tumour or malignancy	21	2.9%
Hemiplegia	16	2.3%
Peripheral vascular diseases	11	1.6%
Dementia	9	1.3%
Liver disease	4	0.6%
AIDS/HIV	2	0.3%
Rheumatological disease	1	0.1%
**Hospital admission due to COPD in the past two years**		
Yes	263	37.3%
**Number of hospital admissions in the past two years**		
Zero	442	62.8%
1	147	20.9%
2	70	9.9%
3	21	3.0%
4	10	1.4%
5	8	1.1%
6	4	0.6%
7	2	0.3%
**ICU hospital admission due to COPD in the past two years**		
Yes	84	12.4%
**Number of ICU admissions in the past two years** (n = 702)		
Zero	615	87.6%
1	66	9.4%
2	16	2.3%
3	4	0.6%
4	1	0.1%
**Long-term non-COPD Medications**		
Antihypertensive	288	40.9%
Oral antidiabetic agents or insulin	279	39.6%
Proton pump inhibitors	190	27.0%
Anti-dyslipidemic	182	25.9%
Antiplatelets	151	21.4%
CHF medications	76	10.8%
Antipsychotic and antidepressants	54	7.7%
Osteoporosis drugs	51	7.2%
Anticoagulants	45	6.4%
Proton pump inhibitors (PPI) and Anti-histamine (H2 blockers)	8	1.1%

Greater a little over one-third of the patients were hospitalized due to COPD in the past two years. Around 12.4% of the hospitalized patients were admitted to the ICD due to COPD over the past two years.

### Long-term COPD medications

The most commonly used long-term controller medications were LAMA, LABA, and ICS, accounting for 80.4%, 59.3%, and 55.3%, respectively. As-needed SABA was prescribed for the majority of patients (90.8%) to relieve symptoms attacks, Table 2.

**Table 2:** Medications use history

Variable	Frequency	Percentage
**SABA**	640	90.8%
**LAMA**	567	80.4%
**LABA**	418	59.3%
**ICS**	390	55.3%
**Systematic corticosteroids**	139	19.7%
**Home oxygen and BIPAP**	55	7.8%
**Home oxygen**	37	5.2%

### Predictors of COPD ICU admissions

Logistic regression analysis identified that older age (65 years and over), smoking, receiving home care, and having specific comorbidities (Hypertension, Diabetes mellitus, Ischemic heart disease, Obstructive sleep apnea syndrome, Heart failure, stroke, and Tumour or malignancy) were predictors of ICU admissions among patients with COPD. Details of the logistic regression analysis are listed in the Table 3.

**Table 3:** Predictors of COPD hospital admissions

Variable	Odds ratio (95% confidence interval)	p-value
**Gender**		
Females (Reference group)	1.00	
Males	1.05 (0.77–1.43)	0.770
**Mean age**		
Below 65 years old	1.00	
65 years and over	1.92 (1.41–2.62)	≤ 0.001
**Mean Body Mass Index (BMI)**		
Below 29.2 kg/cm^2^	1.00	
29.2 kg/cm^2^ and over	1.12 (0.82–1.52)	0.489
**Smoking status**		
Non-smoker (Reference group)	1.00	
Ex-smoker	0.57 (0.41–0.78)	≤ 0.001
Current smoker	1.60 (1.17–2.19)	0.003
**Receive home care**		
No (Reference group)	1.00	
Yes	6.32 (3.32–12.01)	≤ 0.001
**Comorbidities**		
Hypertension	1.98 (1.45–2.71)	≤ 0.001
Diabetes mellitus	1.42 (1.04–1.93)	0.027
Gastroesophageal disease (GERD)	2.81 (1.99–3.96)	≤ 0.001
Ischemic heart disease	3.22 (2.19–4.75)	≤ 0.001
Obstructive sleep apnoea syndrome	2.14 (1.38–3.33)	≤ 0.001
Heart failure	5.16 (2.93–9.10)	≤ 0.001
Chronic kidney disease	1.75 (0.92–3.31)	0.087
stroke	4.51 (2.20–9.26)	≤ 0.001
Connective tissue diseases	0.46 (0.17–1.25)	0.128
Tumour or malignancy	3.12 (1.03–9.40)	0.044
Hemiplegia	2.22 (0.82–6.02)	0.119
Peripheral vascular diseases	0.63 (0.17–2.40)	0.498

## Discussion

The existing body of research presents a range of prevalence rates for ICU admissions related to AECOPD requiring hospitalization, spanning from 2 to 19% [23]. Furthermore, the in-hospital mortality rate for these patients falls within the range of 20–40%, while the risk of re-hospitalization for subsequent severe events stands at 18% among AECOPD cases admitted to the ICU [23]. Our study showed that 12.4% of hospitalized COPD exacerbation patients required transfer and admission to the ICU for higher levels of care. This compares similarly to a study published in Spain, where the ward-to-ICU transfer among the hospitalized COPD patients was about 11.9% [28]. However, Berenyi et al. conducted a large nation-wide based study in Australia and New Zealand which showed that 23.1% of their hospitalized COPD cohort was transferred to the ICU [29]. This in-between studies difference can be related to the fact that later study has included a larger number of patients, probable different ICU admission criteria, and heterogenous COPD phenotypes likely explained by the higher proportion of patients having acute on chronic hypercapnic respiratory failure and higher indices for their severity of disease on presentation as reported in their acute physiology, age, and chronic health evaluation (APACHE) score.

A step-up approach to managing inpatient COPD exacerbation may occasionally require a transfer to the ICU to provide a specialized management for impending or established respiratory failure in conjunction with pharmacotherapy. Such a patient population have a high rate of in-hospital mortality ranging from 17 to 24% [30–32]. Thus, it remains essential to stay vigilant and to identify certain clinical parameters to predict which patients require close monitoring.

We found that age can potentially predict ward-to-ICU transfer in those who are 65 years and older which is consistent with others’ reports that found that COPD patients who were admitted to the ICU were often elderly exceeding the age of 65 and had poor outcomes [33–35].

It is not surprising that current/ex-smokers status was associated with higher rate of transfer to ICU which can potentially be explained by increasing the susceptibility to pulmonary infections and attenuating the immune host system responses [36]. Additionally, smoking has been associated with prolonged mechanical ventilation duration, and ICU length of stay [37]. Previous literature have documented that smoking cessation is associated with lower mortality rate among patients with COPD [38].

The presence of obstructive sleep apnea (OSA) in patients with COPD is referred to as “overlap syndrome”, with an estimated prevalence of 10–15%., which compares similarly to the OSA prevalence among general population [39]. In our study, we found a similar prevalence of 13%. Previously published works have shown that lack of treatment for OSA was associated with a higher risk of mortality and severe COPD exacerbation [40, 41]. Our study substantiated that this cohort of overlap patients may require a higher level of care during their current admission.

Extra-pulmonary comorbidities remain an area of interest in understanding COPD phenotyping and attempting to manage those patients in a multi-disciplinary fashion; however, there is a paucity of data regarding their implications on short-term outcomes during an index in-hospital admission [42]. Both cardiovascular and Stroke diseases share a common risk factor, which is smoking. It is worth noting that cardiovascular comorbid conditions are associated with poor outcome measures in COPD patients. Papaioannou et al. prospectively analyzed 609 COPD patients who were admitted with pulmonary exacerbation and found that cardiovascular disease, systemic hypertension and diabetes mellitus can be predictors of future exacerbation and re-hospitalizations [43]. The co-existence of congestive heart failure cannot be under-estimated; a recent systematic review has concluded that CHF was associated with a 61% higher hazard for mortality when compared to COPD patients without CHF [44]. Our results have confirmed that systemic hypertension, DM, IHD, and CHF are predictors for poor short-term in-patient outcomes; therefore, these factors should be taken into account when managing ward patients and to give clinicians a lower threshold in escalating care to ICU level, if needed. In addition, previous study found that indication for oxygen supplementation was a significant predictor for longer length of stay [45]. In terms of cerebrovascular diseases, Lekoubou et al. found that about 12% of patients with stroke had a concomitant COPD diagnosis, on the other hand, our study only showed a prevalence of 5.4%; this difference is likely due to our smaller sample size [46]. One of the possible explanations for admission to the ICU in stroke/COPD is the potential aspiration and swallowing dysfunction risk which can precipitate an acute exacerbation and pneumonia; stroke patients with COPD were evaluated by a videofluoroscopic swallowing study and were found to have a statistically significant risk of aspiration [47]. Zhang et al. found that patients with concomitant COPD and stroke were two times more likely to have pulmonary infections compared to patients without COPD [48]. This Speech-Language-Pathology area requires further clinical research to provide better understanding of the basic science as well as the clinical outcomes and interventions.

The presence of COPD in critically ill patients is an independent risk factor for mortality and morbidity and is associated with prolonged mechanical ventilation and prolonged weaning [49]. Patients hospitalized with acute exacerbations of chronic obstructive pulmonary disease have poor outcomes and poor short- and long-term survival [31]. The World health organization (WHO) ranked COPD as the third leading cause of death worldwide, causing 3.23 million deaths in 2019 [50]. However, enough data on the causes of ward-COPD patients transfer to the ICU remain unavailable specifically in Saudi Arabia and the Middle East region, and we urge future studies to focus such outcomes.

## Strength and weaknesses

Our study has multiple strengths. Firstly, it is the first study in the region to elaborate on the association of certain pulmonary and non-pulmonary predictors for ICU transfer in patients who were admitted to the ward. Secondly, we could not find a publication in the literature that correlated these parameters with admission to higher acuity settings. Lastly, our work can serve as a validation model to test these predictors on a large-scale dataset. Nevertheless, we acknowledge certain limitations. The retrospective nature of our study may have introduced certain selection bias in excluding patients whom we did not have immediate access to their spirometry data if were done outside given the different electronic medical records used by different hospitals. Also, we did not have immediate access to assess medications compliance and inhalers techniques prior to admission since we receive a large number of patients from the entire region.

## Conclusion

Our study found that a step-up approach to inpatient COPD management requires admission to the ICU in 12.4%. Certain clinical parameters were strongly predictive of this, including age, smoking status, cardiovascular, and stroke. Further clinical research is needed to provide a validated model that can be incorporated into clinical practice to monitor this patient population during their admission and identify at-risk individuals for early transfer to higher acuity settings and intensive care units.

## Data Availability

All data generated or analysed during this study are included in this published article.

## References

[1] Singh D et al. Global strategy for the diagnosis, management, and prevention of chronic obstructive lung Disease: the GOLD science committee report 2019. Eur Respir J. 2019;53(5). 10.1183/13993003.00164-2019 30846476

[2] Decramer M, Janssens W, Miravitlles M. Chronic Obstructive Pulmonary Disease. The Lancet. 2012;379(9823):1341–51. 10.1016/S0140-6736(11)60968-9 PMC7172377 22314182

[3] Forey BA, Thornton AJ, Lee PN. Systematic review with meta-analysis of the epidemiological evidence relating Smoking to COPD, Chronic Bronchitis and Emphysema. BMC Pulm Med. 2011;11(1):1–61. 21672193 10.1186/1471-2466-11-36 PMC3128042

[4] Confalonieri M, et al. Chronic Obstructive Pulmonary Disease Definition: is it time to incorporate the Concept of failure of lung regeneration? Am J Respir Crit Care Med. 2023;207(3):366–7. 36174210 10.1164/rccm.202208-1508LE PMC9896632

[5] Halbert R, et al. Global burden of COPD: systematic review and meta-analysis. Eur Respir J. 2006;28(3):523–32. 16611654 10.1183/09031936.06.00124605

[6] Adeloye D et al. Global and regional estimates of COPD prevalence: systematic review and meta–analysis. J Global Health. 2015;5(2). 10.7189/jogh.05-020415 PMC4693508 26755942

[7] Varmaghani M, et al. Global prevalence of Chronic Obstructive Pulmonary Disease: systematic review and meta-analysis. East Mediterr Health J. 2019;25(1):47–57. 30919925 10.26719/emhj.18.014

[8] Blanco I et al. Geographic distribution of COPD prevalence in the world displayed by Geographic Information System maps. Eur Respir J. 2019;54(1). 10.1183/13993003.00610-2019 31000678

[9] Chick DA, Van Harrison GP et al. R,. *Chronic Obstructive Pulmonary Disease*. 2020; Available from: https://www.ncbi.nlm.nih.gov/books/NBK568512/.

[10] O’Donnell DE. Hyperinflation, dyspnea, and exercise intolerance in chronic obstructive pulmonary disease. Proc Am Thorac Soc. 2006;3(2):180–184. 10.1513/pats.200508-093DO 16565429

[11] Opitz B, et al. Innate immune recognition in infectious and noninfectious Diseases of the lung. Am J Respir Crit Care Med. 2010;181(12):1294–309. 20167850 10.1164/rccm.200909-1427SO

[12] Decramer M, et al. COPD as a lung Disease with systemic consequences–clinical impact, mechanisms, and potential for early intervention. COPD: J Chronic Obstr Pulmonary Disease. 2008;5(4):235–56. 10.1080/15412550802237531 18671149

[13] Agarwal AK, Brown RA. BD. *Chronic Obstructive Pulmonary Disease*. 2023; Available from: https://www.ncbi.nlm.nih.gov/books/NBK559281/.

[14] Lau A-W, Yam L-C, Poon E. Hospital re-admission in patients with acute exacerbation of Chronic Obstructive Pulmonary Disease. Respir Med. 2001;95(11):876–84. 11716201 10.1053/rmed.2001.1180

[15] Njoku CM, et al. Risk factors and associated outcomes of hospital readmission in COPD: a systematic review. Respir Med. 2020;173:105988. 33190738 10.1016/j.rmed.2020.105988

[16] Mannino DM, et al. Prevalence and outcomes of Diabetes, Hypertension and Cardiovascular Disease in COPD. Eur Respir J. 2008;32(4):962–9. 18579551 10.1183/09031936.00012408

[17] Doll H, Miravitlles M. Health-related QOL in acute exacerbations of Chronic Bronchitis and Chronic Obstructive Pulmonary Disease: a review of the literature. PharmacoEconomics. 2005;23:345–63. 15853435 10.2165/00019053-200523040-00005

[18] Soler-Cataluña J, et al. Severe acute exacerbations and mortality in patients with Chronic Obstructive Pulmonary Disease. Thorax. 2005;60(11):925–31. 16055622 10.1136/thx.2005.040527 PMC1747235

[19] Beeh KM, et al. Characterisation of exacerbation risk and exacerbator phenotypes in the POET-COPD trial. Respir Res. 2013;14(1):1–8. 24168767 10.1186/1465-9921-14-116 PMC3833311

[20] Hurst J. Evaluation of COPD longitudinally to identify predictive surrogate endpoints (ECLIPSE) investigators: susceptibility to exacerbation in Chronic Obstructive Pulmonary Disease. N Engl J Med. 2010;363:1128–38. 20843247 10.1056/NEJMoa0909883

[21] Lagoe RJ, Johnson PE, Murphy MP. Inpatient hospital Complications and lengths of stay: a short report. BMC Res Notes. 2011;4(1):1–5. 21545741 10.1186/1756-0500-4-135 PMC3098808

[22] Stone K, et al. A systematic review of the prediction of hospital length of stay: towards a unified framework. PLOS Digit Health. 2022;1(4):e0000017. 36812502 10.1371/journal.pdig.0000017 PMC9931263

[23] Prediletto I, Giancotti G, Nava S. COPD Exacerbation: why it is important to avoid ICU admission. J Clin Med. 2023;12(10). 10.3390/jcm12103369 PMC10218914 37240474

[24] Alaithan AM, et al. Chronic Obstructive Pulmonary Disease: hospital and intensive care unit outcomes in the Kingdom of Saudi Arabia. Int J Chron Obstruct Pulmon Dis. 2012;7:819–23. 23269866 10.2147/COPD.S37611 PMC3529632

[25] Hospital MoHANS. *Al Noor Specialist Hospital*. 2023 [cited 2023 2023]; Available from: http://nsh.med.sa/Pages/AboutUs.aspx#.

[26] Rabe KF, et al. Global strategy for the diagnosis, management, and prevention of Chronic Obstructive Pulmonary Disease: GOLD executive summary. Am J Respir Crit Care Med. 2007;176(6):532–55. 17507545 10.1164/rccm.200703-456SO

[27] Naing NN. Determination of sample size. Malays J Med Sci. 2003;10(2):84–6. 23386802 PMC3561892

[28] Marcos PJ, et al. Relationship between severity classification of Acute Exacerbation of Chronic Obstructive Pulmonary Disease and clinical outcomes in hospitalized patients. Cureus. 2017;9(1):e988. 28265524 10.7759/cureus.988 PMC5323027

[29] Berenyi F, et al. Characteristics and outcomes of critically Ill patients with Acute Exacerbation of Chronic Obstructive Pulmonary Disease in Australia and New Zealand. Ann Am Thorac Soc. 2020;17(6):736–45. 32135066 10.1513/AnnalsATS.201911-821OC

[30] Ongel EA, et al. How do COPD comorbidities affect ICU outcomes? Int J Chron Obstruct Pulmon Dis. 2014;9:1187–96. 25378919 10.2147/COPD.S70257 PMC4207568

[31] Seneff MG, et al. Hospital and 1-year survival of patients admitted to intensive care units with acute exacerbation of Chronic Obstructive Pulmonary Disease. JAMA. 1995;274(23):1852–7. 7500534

[32] Ai-Ping C, Lee KH, Lim TK. -hospital and 5-year mortality of patients treated in the ICU for acute exacerbation of COPD: a retrospective study. Chest. 2005;128(2):518–24. 16100133 10.1378/chest.128.2.518

[33] Gadre SK, et al. Acute Respiratory Failure requiring mechanical ventilation in severe Chronic Obstructive Pulmonary Disease (COPD). Med (Baltim). 2018;97(17):e0487. 10.1097/MD.0000000000010487 PMC5944543 29703009

[34] Brown H, et al. Factors associated with hospital mortality in critically ill patients with exacerbation of COPD. Int J Chron Obstruct Pulmon Dis. 2018;13:2361–6. 30122916 10.2147/COPD.S168983 PMC6080864

[35] Teixeira C, et al. Patients admitted to the ICU for acute exacerbation of COPD: two-year mortality and functional status. J Bras Pneumol. 2011;37(3):334–40. 21755188 10.1590/s1806-37132011000300009

[36] Bello S, et al. Tobacco Smoking increases the risk for death from pneumococcal Pneumonia. Chest. 2014;146(4):1029–37. 24811098 10.1378/chest.13-2853

[37] Alroumi F, et al. The impact of smoking on patient outcomes in severe sepsis and septic shock. J Intensive Care. 2018;6:42. 30065844 10.1186/s40560-018-0312-x PMC6064183

[38] Doo JH, et al. Smoking cessation after diagnosis of COPD is associated with lower all-cause and cause-specific mortality: a nationwide population-based cohort study of South Korean men. BMC Pulm Med. 2023;23(1):237. 37394482 10.1186/s12890-023-02533-1 PMC10316560

[39] Soler X, et al. High prevalence of obstructive sleep apnea in patients with moderate to severe Chronic Obstructive Pulmonary Disease. Ann Am Thorac Soc. 2015;12(8):1219–25. 25871443 10.1513/AnnalsATS.201407-336OC PMC5466175

[40] Marin JM, et al. Outcomes in patients with Chronic Obstructive Pulmonary Disease and obstructive sleep apnea: the overlap syndrome. Am J Respir Crit Care Med. 2010;182(3):325–31. 20378728 10.1164/rccm.200912-1869OC

[41] Stanchina ML, et al. Impact of CPAP use and age on mortality in patients with combined COPD and obstructive sleep apnea: the overlap syndrome. J Clin Sleep Med. 2013;9(8):767–72. 23946706 10.5664/jcsm.2916 PMC3716667

[42] Cavaillès A, et al. Comorbidities of COPD. Eur Respir Rev. 2013;22(130):454–75. 24293462 10.1183/09059180.00008612 PMC9639181

[43] Papaioannou AI, et al. Cardiovascular comorbidities in hospitalised COPD patients: a determinant of future risk? Eur Respir J. 2015;46(3):846–9. 25882807 10.1183/09031936.00237014

[44] Axson EL, et al. Hospitalisation and mortality in patients with comorbid COPD and Heart Failure: a systematic review and meta-analysis. Respir Res. 2020;21(1):54. 32059680 10.1186/s12931-020-1312-7 PMC7023777

[45] Lüthi-Corridori G et al. Predictors of length of Stay, Mortality and Rehospitalization in COPD patients: a retrospective cohort study. J Clin Med. 2023;12(16). 10.3390/jcm12165322 PMC10455093 37629364

[46] Lekoubou A, Ovbiagele B, eNeurologicalSci. 2017. 6: p. 21–24. 10.1016/j.ensci.2016.11.007 PMC5168723 28018982

[47] Park GW, et al. Effect of Chronic Obstructive Pulmonary Disease on swallowing function in Stroke patients. Ann Rehabil Med. 2015;39(2):218–25. 25932418 10.5535/arm.2015.39.2.218 PMC4414968

[48] Zhang S, Liling QZ, Wei et al. Hospitalized Prognosis of Ischemic Stroke with COPD: A Propensity Score Matching Study, 22 October 2021, PREPRINT (Version 1) available at Research Square. 2021.

[49] Funk GC, et al. Prevalence and prognosis of COPD in critically ill patients between 1998 and 2008. Eur Respir J. 2013;41(4):792–9. 23018915 10.1183/09031936.00226411

[50] World Health Organization. Chronic obstructive pulmonary disease (COPD). 2023 [cited 2023 July 2023]; Available from: https://www.who.int/news-room/fact-sheets/detail/chronic-obstructive-pulmonary-disease-(copd).

