# Correction: The rate of ward to intensive care transfer and its predictors among hospitalized COPD patients, a retrospective study in a local tertiary center in Saudi Arabia

**DOI:** 10.1186/s12890-024-03285-2

**Published:** 2024-10-04

**Authors:** Abdallah Y. Naser, Mohammad Saleh Dairi, Hassan Alwafi, Deema Sami Ashoor, Sami Qadus, Abdulelah M. Aldhahir, Abdullah A. Alqarni, Wael Aly Elrefaey, Sultan Qanash, Waleed Hafiz, Jaber S. Alqahtani, Rakan Ekram, Amjad Abuirmeileh, Anan S. Jarab, Omaima Ibrahim Badr

**Affiliations:** 1 Department of Applied Pharmaceutical Sciences and Clinical Pharmacy, Faculty of Pharmacy, Isra University, Amman, Jordan; 2 Department of Medicine, College of Medicine, Umm Al-Qura University, Makkah 21955, Saudi Arabia; 3 Pharmacology and Toxicology Department, Faculty of Medicine, Umm Al-Qura University, Makkah, 21955 Saudi Arabia; 4 Department of Pharmacy, Faculty of health sciences, American University of Madaba, Madaba, Jordan; 5 Respiratory Therapy Department, Faculty of Applied Medical Sciences, Jazan University, Jazan, Saudi Arabia; 6 Department of Respiratory Therapy, Faculty of Medical Rehabilitation Sciences, King Abdulaziz University, Jeddah, Saudi Arabia; 7 Department of Pulmonary Medicine, Al Noor Specialist Hospital, Mecca, Saudi Arabia; 8 Department of Internal Medicine, National Guard Hospital, Jeddah, Saudi Arabia; 9 Department of Respiratory Care, Prince Sultan Military College of Health Sciences, Dammam, Saudi Arabia; 10 School of Public Health and Health Informatics, Umm Al-Qura University, Mecca, Saudi Arabia; 11 Faculty of Pharmacy, Middle East University, Amman, Jordan; 12 College of Pharmacy, AL Ain University, P.O. Box 112612, Abu Dhabi, United Arab Emirates; 13 AAU Health and Biomedical Research Center, Al Ain University, P.O. Box 112612, Abu Dhabi, United Arab Emirates; 14 Department of Clinical Pharmacy, Faculty of Pharmacy, Jordan University of Science and Technology, Irbid, 22110 Jordan; 15 Department of Chest Medicine, Faculty of Medicine, Mansoura University, Mansoura, 35516 Egypt

**Correction: BMC Pulmonary Medicine (2023) 23:464**

10.1186/s12890-023-02775-z

Following publication of the original article, it came to the attention of the authors that the affiliation of the second author, Mohammad Saleh Dairi, was incorrect. It has since been updated in the article; please refer to the article for the updated, correct affiliations information. The authors thank you for reading this erratum and apologize for any inconvenience caused.

